# ^68^Ga-DOTA-TATE PET/CT improves accuracy and guides management in multiple endocrine neoplasia type 1 (MEN-1) patients with suspected duodeno-pancreatic neuroendocrine tumours

**DOI:** 10.1530/EO-25-0060

**Published:** 2025-09-17

**Authors:** Kalyan Vamshi Vemulapalli, Kalyan Mansukhbhai Shekhda, Gowri Ratnayake, Gopinath Gnanasegaran, Ann-Marie Quigley, Aimee R Hayes, Bernard Khoo, Dalvinder Mandair, Christos Toumpanakis, Martyn Caplin, Ashley B Grossman, Shaunak Navalkissoor

**Affiliations:** ^1^ Nuclear Medicine Department, Royal Free Hospital, London, UK; ^2^ Academic Foundation Programme, University College London, London, UK; ^3^ Neuroendocrine Tumour Unit, ENETS Centre of Excellence, Royal Free Hospital, London, UK

**Keywords:** ^68^Ga-DOTA-TATE PET/CT, MEN-1, duodeno-pancreatic neuroendocrine tumours

## Abstract

**Purpose:**

To evaluate the added benefit and accuracy of ^68^Ga-DOTA-TATE PET/CT scans in detecting duodeno-pancreatic neuroendocrine tumours (dpNETs) compared to conventional cross-sectional imaging with CT or MRI scans in patients with multiple endocrine neoplasia type 1 (MEN-1), and whether the results from the ^68^Ga-DOTA-TATE PET/CT produce a change in management plans for patients with MEN-1 and dpNETs.

**Methods:**

A retrospective analysis was performed comparing the initial ^68^Ga-DOTA-TATE PET/CT to the respective contemporary CT or MRI imaging in patients with MEN-1 under the care of a tertiary neuroendocrine centre. Imaging and electronic patient records were analysed to identify treatment plans and the records of multidisciplinary team discussions.

**Results:**

In total, 85% (*n* = 39/46) of patients with MEN-1 had a ^68^Ga-DOTA-TATE PET/CT study in the electronic patient record; 23 of those with duodeno-pancreatic lesions detected also had contemporaneous contrast-enhanced CT scans, while 18 had MRI scans. ^68^Ga-DOTA-TATE PET/CT detected a total of 47 pancreatic lesions compared to 25 on CT, while ^68^Ga-DOTA-TATE PET/CT detected 32 pancreatic lesions compared to 25 on MRI. There were no duodenal lesions detected on conventional CT or MRI, but in comparison to CT and MRI, ^68^Ga-DOTA-TATE PET/CT detected eight and one duodenal lesions respectively. While ^68^Ga-DOTA-TATE PET/CT detected more liver metastases compared to CT (*n*: 31 vs 21) and similar numbers compared to MRI (*n*: 11 vs 11), these differences were not statistically significant. As a result of findings on ^68^Ga-DOTA-TATE PET/CT, a change of management was indicated in 69% (*n* = 27/39) of patients. Of these, 14 patients were offered somatostatin analogues (SSTA), eight patients were offered surgical intervention, three patients were offered peptide receptor radionuclide therapy, and one patient was offered ablation of liver metastases.

**Conclusions:**

In patients with MEN-1, ^68^Ga-DOTA-TATE PET/CT was shown to detect a greater number of duodeno-pancreatic lesions compared to conventional cross-sectional CT or MRI imaging. Management plans were changed in most patients following their initial ^68^Ga-DOTA-TATE PET/CT. Therefore, we suggest that somatostatin receptor-targeted PET/CT scans should be an integral part of the investigation of patients with MEN-1 for staging of suspected duodeno-pancreatic NETs.

## Introduction

Multiple endocrine neoplasia type 1 (MEN-1) is an autosomal dominant hereditary syndrome caused by a mutation in the MEN-1 tumour suppressor gene (menin) located on chromosome 11q13 (1). The annual incidence of MEN-1 is estimated to be between 1 in 10,000 and 1 in 100,000 (2, 3, 4). The disease is characterised by the occurrence of two or more endocrine tumours in the parathyroid glands, the anterior pituitary gland, and neuroendocrine tumours (NETs) in the islets of the pancreas (4). Further manifestations may also include NETs in the thymus, bronchus, and adrenal glands, as well as various skin manifestations (4). Based on regular screening and advanced imaging techniques, the lifetime risk of duodeno-pancreatic NETs (dpNETs), including functional and non-functional tumours in the setting of MEN-1, is >90% (5).

The most common direct cause of mortality in MEN-1 is due to malignant dpNETs and thymic NETs (3, 6, 7, 8). Therefore, once a clinical and/or genetic diagnosis is established, active surveillance through regular clinical follow-up, biochemical testing, and surveillance imaging is recommended by clinical practice guidelines (3).

Along with more conventional cross-sectional imaging in the form of computerised tomography (CT) and magnetic resonance imaging (MRI), positron emission tomography (PET) imaging is also employed for detecting NETs in patients with MEN-1 (9, 10). Most NETs tend to express somatostatin receptors (SSTR), and thus SSTR PET scans that can take advantage of this can be employed (11, 12, 13). A positron-emitting isotope such as ^68^Ga can be paired with a somatostatin analogue to help visualise the locations of NETs on PET imaging. Examples of these radioligands include ^68^Ga-DOTA-TATE, ^68^Ga-DOTA-TOC, and ^68^Ga-DOTA-NOC (11). Although no head-to-head studies are available, a meta-analysis of ten studies found that ^68^Ga-DOTA-TATE PET demonstrated higher diagnostic sensitivities and specificities compared with ^68^Ga-DOTA-TOC PET in patients with NETs (14), and thus this ^68^Ga-tracer-based PET/CT has become the gold standard in the staging and management of well-differentiated neuroendocrine tumours (3, 15, 16, 17, 18, 19). It has been shown to significantly impact the management of patients with NETs (12, 13), but the clinical utility of using ^68^Ga-DOTA-TATE PET/CT in staging, screening, and active surveillance, specifically in patients with MEN-1, is still relatively unclear.

Therefore, the aim of this study was to determine whether ^68^Ga-DOTA-TATE PET/CT is more accurate at detecting and/or staging dpNETs in patients with MEN-1 compared to conventional cross-sectional CT or MRI imaging, and whether the results from the ^68^Ga-DOTA-TATE PET/CT would result in a change of management plans for patients with MEN-1 and dpNETs.

## Methods

Consecutive patients who were under the care of the neuroendocrine tumour (NET) Unit at the Royal Free Hospital, London, United Kingdom, between 2008 and 2024, with a positive clinical and/or genetic diagnosis of MEN-1, were screened for inclusion in this retrospective case series.

At our centre, all patients with MEN-1 are followed up regularly for clinical examination, cross-sectional CT/MRI every 6–12 months, and/or biochemical analysis with a fasting gut hormone profile as per current guidelines. During follow-up, if there is a clinical and/or radiological suspicion of a dpNET, their cases are discussed in a multidisciplinary tumour board meeting (which involves endocrinologists, nuclear medicine physicians, a NET specialist pathologist, gastroenterologist and oncologist, and appropriate surgeons). After discussion in the tumour board meeting, further investigations and/or management plans are made. Further investigations include functional imaging such as ^68^Ga-DOTA-TATE PET/CT, and invasive procedures such as endoscopic ultrasound (EUS). Indications for ^68^Ga-DOTA-TATE PET/CT was for staging, restaging of dpNETs, staging for evaluation for possible PRRT, evaluation of suspected gastrinoma, and investigation of suspected dpNET.

At our centre, EUS is performed by experienced pancreato-biliary physicians and is usually carried out for clinical suspicion of a dpNET which is not detected on conventional imaging modalities, to confirm the histological diagnosis in patients with suspicion of dpNET, before surgical intervention as a guide to the surgical procedure in patients with confirmed dpNET, and in patients with locoregional metastases to confirm and/or guide surgical intervention.

Inclusion criteria for this study included: i) patients diagnosed with MEN-1 either through genetic or clinical criteria (3), ii) who had a ^68^Ga-DOTA-TATE PET/CT scan on their hospital record, iii) who had a contemporaneous CT or MRI scan ideally within 6 months preceding or following the ^68^Ga-DOTA-TATE PET/CT scan, and iv) a documented plan of action following the results of these studies.

An analysis was then performed comparing the images and reports of the initial ^68^Ga-DOTA-TATE PET/CT to the contemporaneous CT or MRI imaging. All images were re-read by consensus for the purpose of this study by two experienced nuclear medicine physicians expert in SSTR-PET (somatostatin receptor-positron emission tomography) and NETs. There was no blinding between the imaging studies when making these comparisons. The number and location of lesions detected by each imaging modality were collated and compared. The electronic patient record, imaging, and summaries of multidisciplinary team (MDT) discussions were also examined to determine the outcomes and follow-up of patients following their investigative studies.

Conventional cross-sectional radiological imaging was defined as either CT scanning or MRI scanning. Where available, validation of the findings on ^68^Ga-DOTA-TATE PET/CT was carried out by comparing with histology and cytology, considered the ‘gold standard’. Where histology was not available, comparisons were made with clinical follow-up, biochemical evaluation (fasting gut hormone profile, if available), conventional imaging or EUS, or the outcome of the MDT meeting discussion.

### Imaging evaluation details

Studies were carried out using the protocols below. All conventional studies were re-reported by NET-experienced radiologists, and the ^68^Ga-DOTA-TATE PET/CT by nuclear medicine physicians with expertise in NETs.

#### CT protocol

Triple-phase CT-CAP (chest, abdomen, pelvis) was performed, which comprised a non-contrast study and two contrast-enhanced studies. These contrast-enhanced studies comprised an arterial phase (30 s after contrast administration) and a porto-venous phase (70 s after contrast administration).

#### MRI protocol

A multiparametric MRI (mpMRI) scan of the abdomen and pelvis was performed using a Siemens MAGNETOM 1.5T, which included an arterial phase study, porto-venous phase study, and diffusion-weighted images was made (*b* = 50,600 and 1,000 s/mm^2^).

#### ^68^Ga-DOTA-TATE PET/CT protocol

Approximately 150MBq of ^68^Ga-DOTA-TATE was administered intravenously, with PET/CT imaging performed on Siemens Biograph mCT 128 from the vertex to thigh between 45 and 70 min following administration of the tracer. The base reconstruction was Siemens ‘TrueX’ OSEM (2i21s) iterative reconstruction with time-of-flight and point-spread function. The concomitant CT was low-dose, non-contrast.

### Statistics

Data collection was performed using Microsoft Excel® (Windows). Further data preparation and statistical analyses were performed using SPSS (IBM SPSS version 23). Comparison of the number of lesions detected by ^68^Ga-DOTA-TATE PET/CT, MRI imaging, and CT imaging was done using the Mann–Whitney U test with a significance level of 0.05. In all the comparisons, ‘exact significance’ (Exact Sig.) was taken into consideration as sample size was <20 in either group, except in the comparison of ^68^Ga-DOTA-TATE PET/CT with CT imaging for detection of pancreatic lesions as in both groups the sample size was >20. When comparing the number of pancreatic lesions, duodenal lesions, locoregional metastases, liver metastases, and distant metastases detected by ^68^Ga-DOTA-TATE PET/CT with MRI or CT, the null hypothesis was deemed to be that there would be no difference between the number of lesions detected on ^68^Ga-DOTA-TATE PET/CT compared to either MRI or CT. The alternate hypothesis was that ^68^Ga-DOTA-TATE PET/CT would detect significantly more lesions compared to MRI or CT. Patients with a histologically confirmed diagnosis of NETs were included in the calculation for sensitivity, specificity, diagnostic accuracy, positive predictive value, and negative predictive value of ^68^Ga-DOTA-TATE PET/CT for the diagnosis of NETs. The comparison of ^68^Ga-DOTA-TATE PET/CT was done with the gold standard, e.g. histologically confirmed diagnosis of NETs. The confidence interval was 95%.

Since this study was a retrospective evaluation of service/audit, ethical approval was not required under the UK Policy Framework for Health and Social Care Practice (audit registration number: CFHGCS145).

## Results

The baseline demographic details of the patients in the study cohort are summarised in Table 1. Overall, 39 out of 49 patients with MEN-1 met the previously noted inclusion criteria. The reason for all ten of the excluded patients was a lack of a recorded ^68^Ga-DOTA-TATE PET/CT. The mean age was 54 ± 13 years, and 36% (*n* = 14/39) of patients were female. Of the 39 patients included, 95% (*n* = 37) had primary hyperparathyroidism, 97% (*n* = 38) had pancreatic lesions, and 46% (*n* = 18) had pituitary lesions. Around half of the patients (*n* = 19) had a metastatic dpNET (26% locoregional metastases, 21% liver metastases, 21% distant metastases). Of the 39 patients, 13 patients (33%) had a history of abdominal surgery before their first recorded ^68^Ga-DOTA-TATE PET/CT. Indications for ^68^Ga-DOTA-TATE PET/CT were for staging of dpNET [*n*: 16 (41%)], restaging of dpNET [*n*: 9 (23%)], staging for evaluation for possible PRRT [*n*: 3 (8%)], evaluation of suspected gastrinoma [*n*: 1 (5%)], and investigation of suspected dpNET [*n*: 10 (26%)]. Most CT scans and MRI scans were performed within a 6-month interval either preceding or following the ^68^Ga-DOTA-TATE PET/CT. The average interval was ±4.7 months for CT scans and ±2 months for MRI.

**Table 1:** Demographic details of patients who met study inclusion criteria.

Demographic variable	*n* = 39
Sex	
Male	25 (64%)
Female	14 (36%)
Age (years)	54 ± 13
Number with genetic testing confirmation	30 (77%)
**Manifestations**	
Primary hyperparathyroidism	37 (95%)
Pituitary tumours	18 (46%)
Pancreatic tumours	38 (97%)
Adrenocortical adenoma	11 (28%)
Thymic tumour	3 (8%)
Bronchial/lung tumour	6 (15%)
**Metastases** *	
Locoregional metastases on ^68^Ga-DOTA-TATE PET/CT	10 (26%)
Present prior to ^68^Ga-DOTA-TATE PET/CT	5 (50%)
New detected on ^68^Ga-DOTA-TATE PET/CT	5 (50%)
Liver metastases on ^68^Ga-DOTA-TATE PET/CT	8 (21%)
Present prior to ^68^Ga-DOTA-TATE PET/CT	8 (100%)
New detected on ^68^Ga-DOTA-TATE PET/CT	0 (0%)
Distant metastases after ^68^Ga-DOTA-TATE PET/CT	8 (21%)
Present prior to ^68^Ga-DOTA-TATE PET/CT	5 (63%)
New detected on ^68^Ga-DOTA-TATE PET/CT	3 (37%)
No metastases	20 (51%)
**Type of duodeno-pancreatic NET**	
Non-functioning	24 (62%)
Insulinoma	3 (8%)
Pancreatic gastrinoma	7 (18%)
Duodenal gastrinoma	4 (10%)
Glucagonoma	1 (3%)
**Grade of NET**	
Grade 1/Ki67 ≤ 2%	15 (38%)
Grade 2/Ki67 3–20%	14 (36%)
Grade 3/Ki67 > 20%	1 (3%)
Normal histology	1 (3%)
No histology performed	3 (8%)
No grade or Ki67 given from histology	5 (13%)
**Prior abdominal surgery**	
Prior duodenopancreatic surgery	
Whipple surgery	1 (3%)
Enucleation of pancreatic head mass and peripancreatic nodes dissection	1 (3%)
Distal pancreatectomy	2 (5%)
Partial pancreatectomy	1 (3%)
Whipple surgery and distal pancreatectomy	1 (3%)
Prior combined duodenopancreatic and other abdominal surgery	
Central pancreatectomy and adrenalectomy	1 (3%)
Distal pancreatectomy and splenectomy	2 (5%)
Prior abdominal surgery other than duodenopancreatic surgery	
Resection of proximal jejunum	1 (3%)
Small bowel resection	1 (3%)
Adrenalectomy only	1 (3%)
Gastrojejunal bypass	1 (3%)
No prior abdominal surgery	26 (67%)

NET, neuroendocrine tumour.

* Two patients had liver and distal metastases together, three patients had liver and local metastases together, and two patients had local and distal metastases together.

A comparison of detection of various lesions (pancreatic lesions, duodenal lesions, locoregional lymph node metastases, liver metastases, distant metastases) on conventional imaging modalities (CT/MRI) and ^68^Ga-DOTA-TATE PET/CT is summarised in Table 2. Pancreatic lesions on ^68^Ga-DOTA-TATE PET/CT were statistically significantly increased compared to conventional CT imaging (47 vs 25, *P* = 0.001). Although ^68^Ga-DOTA-TATE PET/CT scan detected more pancreatic lesions compared to MRI scans, they were not statistically significant (37 vs 25, *P* = 0.116). There were no duodenal lesions detected on conventional CT or MRI, but ^68^Ga-DOTA-TATE PET/CT detected eight and one duodenal lesions, respectively, although this comparison was not statistically significant. Of the total cohort, four patients (10%) ^68^Ga-DOTA-TATE PET/CT detected avid lung lesions, while these lesions were also detected in conventional cross-sectional imaging (CT Thorax). Clinical and radiological features of patients with functional pancreatic NETs and gastrinomas are described in Supplementary Table 1 (see section on Supplementary materials given at the end of the article).

**Table 2:** Summary of number of pancreatic lesions, duodenal lesions, locoregional lymph node metastases, liver metastases, and distant metastases detected by conventional CT or MRI imaging compared to the number of lesions detected by ^68^Ga-DOTA-TATE PET/CT.

	Conventional cross-sectional scan modality	Lesions on conventional scans	Lesions on ^68^Ga-DOTA-TATE PET/CT	Significance (*P*-value)
Pancreatic lesions	CT (*n* = 22)	25	47	0.001*
	MRI (*n* = 15)	25	37	0.116^†^
Duodenal lesions	CT (*n* = 5)	0	8	0.08^†^
	MRI (*n* = 1)	0	1	0.333^†^
Locoregional lymph node metastases	CT (*n* = 7)	8	15	0.165^†^
	MRI (*n* = 2)	3	4	0.667^†^
Liver metastases	CT (*n* = 3)	21	31	1.000^†^
	MRI (*n* = 4)	11	11	1.000^†^
Distant metastases (lung, mediastinal lymph nodes)	CT (*n* = 9)	14	21	0.222^†^
	MRI (*n* = 1)	1	2	1.000^†^

CT, computed tomography; MRI, magnetic resonance imaging.

* Asymp.Sig.; asymptomatic significance.

^†^ Exact Sig.; exact significance.

**Figure 1:**
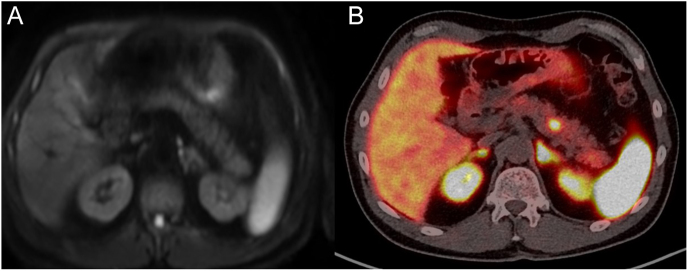
(A) MRI abdomen: difficult to identify pancreatic lesion on MRI. (B) ^68^Ga-DOTA-TATE PET/CT avid pancreatic body lesion.

Of the 39 patients, a total of 15 patients had either functional pancreatic NETs or duodenal NETs (duodenal gastrinoma (*n* = 4), pancreatic gastrinoma (*n* = 7), insulinoma (*n* = 3), glucagonoma (*n* = 1)). Of these patients, a 54-year-old man had a normal MRI of the abdomen but abnormal avidity on ^68^Ga-DOTA-TATE PET/CT in the body of the pancreas (Fig. 1).

The sensitivity, specificity, diagnostic accuracy, positive predictive value, and negative predictive value of ^68^Ga-DOTA-TATE PET/CT compared to EUS plus biopsy are described in Table 3. One of these apparent false positives was still deemed to be of high enough risk to warrant the MDT recommending the patient to start SSTA treatment. For another false positive, while the EUS reported two hypoechoic lesions in the pancreas, the subsequent biopsy was negative. This patient was recommended to have a repeat EUS and biopsy and, in the interim, to start SSTA treatment.

**Table 3:** Sensitivity, specificity, diagnostic accuracy, positive predictive value, negative predictive value of ^68^Ga-DOTA-TATE PET/CT for the diagnosis of neuroendocrine tumour compared to gold standard (histologically confirmed diagnosis)*.

Statistic	Value	95% CI
Sensitivity	100%	100.0%
Specificity	33.33%	0–55%
Positive predictive value	83.33%	68.4–98.2%
Negative predictive value	100.00%	100.00%
Diagnostic accuracy	84%	69.6–98.4%

* True positive (TP): *n* = 20, true negative (TN): *n* = 1, false positive (FP): *n* = 4, false negative: *n* = 0.

Table 4 summarises the outcome from the MDT meetings or following their review of the patients’ ^68^Ga-DOTA-TATE PET/CT images and their reports, along with review of other available investigations. In 20 patients, changes in management were recommended without the need for further investigation. Of the 11 patients who were recommended to have further investigations following their ^68^Ga-DOTA-TATE PET/CT, seven patients had a further change in treatment plans following further investigations. Therefore, a total of 27 patients (69.2%) ultimately had a change in management recommendations following ^68^Ga-DOTA-TATE PET/CT. The details of changes in management are described in Table 4.

**Table 4:** Recommendations by the multidisciplinary team meeting following review of the ^68^Ga-DOTA-TATE PET/CT images and reports in patients with MEN-1.

Management recommendation with ^68^Ga-DOTA-TATE PET/CT results	*n* (total *n* = 39)
Ablation of liver metastasis	1
Further investigations	11
Oesophago-gastroduodenoscopy followed by high doses of PPI	1
EUS followed by surgery (1 distal pancreatectomy, 1 partial pancreatectomy)	2
EUS followed by somatostatin analogue therapy	4
EUS followed by active surveillance	4
Somatostatin analogue therapy	10
Surgery for curative intent	5
Surgery for debulking/palliation	1
Peptide receptor radionuclide therapy (PRRT)	3
Active surveillance	8

PPI, proton pump inhibitor; EUS, endoscopic ultrasound; PRRT, peptide receptor radionuclide therapy.

## Discussion

We investigated the clinical utility of ^68^Ga-DOTA-TATE PET/CT imaging for the diagnosis and management of dpNETs in patients with MEN-1 compared to conventional cross-sectional MRI and CT imaging and overall found that such scanning both increased the detection of lesions and significantly changed management in the majority of patients. The major difference in our study compared to previous studies is that we specifically looked at the actual number of dpNET lesions compared to conventional imaging and invasive methods such as EUS. The results from previous studies were uncertain in terms of whether the duodeno-pancreatic lesions detected on functional imaging were actual dpNETs or metastases. In addition, some of the studies used different molecular agents, e.g. DOTA-TOC, and not all studies evaluated changes in actual management in these patient cohorts. Therefore, we conclude that this imaging modality is invaluable as a non-invasive study in surgical planning and also adds to the literature regarding the value of ^68^Ga-DOTA-TATE PET/CT before surgical/medical management of NETs in patients with MEN-1. The detailed results of previously published studies are described in Supplementary Table 2.

### Usefulness of ^68^Ga-DOTA-TATE PET/CT in screening and diagnosis of metastatic pancreatic and duodenal NET

The results from our study revealed that ^68^Ga-DOTA-TATE PET/CT detected more lesions compared to conventional imaging modalities. In particular, it was found to be useful in detecting additional pancreatic, duodenal, locoregional, and distant metastases, but not liver metastases to a significant extent, as described above. On reviewing previous studies (Supplementary Table 2), functional imaging using ^68^Ga-DOTA-TATE/TOC has been reported to detect additional lesions compared to conventional imaging such as CT/MRI. In a prospective study by Sadowski *et al.* in 2015 (9), the authors compared ^68^Ga-DOTA-TATE PET/CT, ^111^In-pentetreotide SPECT/CT, and triphasic CT scan data in patients with MEN-1. ^68^Ga-DOTA-TATE PET/CT detected additional lesions in 61.5% of patients that were not detected on other imaging modalities. However, the authors did not specify the type of lesions detected more by ^68^Ga-DOTA-TATE PET/CT. Regarding the utility of ^68^Ga-DOTA-TATE PET/CT as an initial screening method, Lastoria *et al.* (10) compared ^68^Ga-DOTA-TATE PET/CT with CECT and EUS in 18 patients with MEN-1 (10). However, the focus of their study was on the pancreas, pituitary, parathyroids, and adrenals, but not on NETs or metastases in other locations. Their results suggested 100% specificity and 100% sensitivity of ^68^Ga-DOTA-TATE PET/CT for pancreatic lesions. This is in contrast to our finding of the sensitivity and specificity of ^68^Ga-DOTA-TATE PET/CT in the detection of dpNET being 100 and 33.33% respectively. While the authors did not draw definitive conclusions, they concluded that ^68^Ga-DOTA-TATE PET/CT could be useful in the initial work-up of patients with MEN-1. In contrast to these studies and our own, two studies by Morgat *et al.* (20) and Albers *et al.* (21), in which the utility of ^68^Ga-DOTA-TOC PET/CT was compared to conventional imaging modalities such as CECT, MRI, and EUS in MEN-1 patients (20, 21), Morgat and colleagues found that while ^68^Ga-DOTA-TOC PET/CT helped identify 15 NETs that were initially not seen on contrast-enhanced CT, there were also 13 NETs that were seen by CT but were not detected by the ^68^Ga-DOTA-TOC PET/CT (20). It is possible that the increased sensitivity shown in our study may be the result of the improved affinity of ^68^Ga-DOTA-TATE for somatostatin receptor subtype-2 versus ^68^Ga-DOTA-TOC, with up to ten times higher binding affinity (11). Therefore, this may impact the difference in clinical utility of these tracers. Albers and colleagues investigated imaging using ^68^Ga-DOTA-TOC PET/CT as a screening tool for MEN-1 patients and reported that clinical management was not changed in 97% of patients; therefore, its use in MEN-1 patient screening was not recommended (21). However, most of the additional lesions that were not seen by ^68^Ga-DOTA-TOC PET/CT were detected by EUS. In addition, ^68^Ga-DOTA-TOC PET/CT was found to detect more lesions than MRI in their study (21). This methodology contrasts with our study, where EUS, a relatively invasive procedure, was not classified as a form of conventional cross-sectional imaging and was reserved for selected patients as described previously or if surgery was being considered. In addition, as noted above, the differences between ^68^Ga-DOTA-TATE and ^68^Ga-DOTA-TOC as tracers may also have had an impact. Kostiainen and colleagues also investigated the usefulness of ^68^Ga-DOTA-NOC PET/CT compared to CT/MRI and found that ^68^Ga-DOTA-NOC PET/CT detected three times as many panNETs which were not visible on conventional imaging modalities such as CT/MRI (22). These results suggest that many lesions, especially duodeno-pancreatic lesions, are being missed by conventional imaging modalities, which could have implications for patient care and prognosis. As the highest cause of mortality in MEN-1 is from duodeno-pancreatic neuroendocrine tumours (3, 4, 6, 8), this is an important aspect to detect, stage, and monitor with the greatest possible accuracy.

### Usefulness of ^68^Ga-DOTA-TATE PET/CT in changing the management of patients with MEN-1

As a result of the ^68^Ga-DOTA-TATE PET/CT, treatment plans were changed in 69% (*n* = 27/39) patients, with immediate changes to management plans without awaiting any further investigations occurring in 51% (*n* = 20/39) patients. Of these 20 patients, the majority were offered SSTAs (*n*: 10), followed by surgery (*n* = 6), PRRT (*n* = 3), and ablation of liver metastases (*n* = 1); 11 patients were offered further investigation, of whom seven patients were offered further treatment (four SSTAs, two surgery, one high dose of PPI). It is worth noting that for these 27 patients in whom a change in management was recommended, the decision could not have been reached without the use of ^68^Ga-DOTA-TATE PET/CT. These findings compare favourably with previous studies described in Supplementary Table 2. In their study, Sadowski and colleagues described that just about one-third of patients had their management plans changed based on ^68^Ga-DOTA-TATE PET/CT findings (9). Froeling *et al.* and Kostiainen *et al.* both reported that about half of their patients had a change in management plans based on ^68^Ga-DOTA-TOC PET/CT and ^68^Ga-DOTA-NOC PET/CT results (22, 23). However, in the study by Froeling *et al.*, the change in management was primarily surgical intervention (none of the patients was offered PRRT or SSTA treatment), in contrast to our study, where the majority of patients had SSTA treatment and PRRT as their change in management plan (23). Similarly, in the study by Kostiainen *et al.,* the majority of patients had been offered surgical intervention (7 out of 12), followed by other systemic therapy (2 out of 12), SSTAs (2 out of 12), and PRRT (1 out of 12) (22). This suggests that ^68^Ga-DOTA-TATE PET/CT may play a key role in aiding NET clinicians and surgeons in determining management plans for patients with MEN-1 and NETs, and therefore its utility cannot be understated. One consideration for clinical implementation would be the availability of ^68^Ga-DOTA-TATE PET/CT for patients. As of 2021, only seven UK centres had the facility to generate and produce ^68^Ga-labelled radiopharmaceuticals (24), of which four are based in London, meaning access would not be equal for all patients living across the UK, and even more so in other countries where facilities for such PET/CT imaging are limited.

### Current guidelines and expert consensus statement regarding use of SSTR (somatostatin receptor) based imaging in management and treatment of MEN-1 and/or NET

Table 5 describes current guidelines for the use of SSTR-based (somatostatin receptor-based) imaging in management and treatment of MEN-1 and/or NETs. The recommendations are unclear as to when SSTR-based imaging should be considered in the management of MEN-1. In addition, several studies have highlighted the superiority of ^68^Ga-tracer based PET/CT when compared to more conventional imaging, including ^18^F-FDG-PET, CT, and MRI, for staging neuroendocrine tumours (25, 26, 27, 28). A recently published recommendation and ‘best practice’ guidelines suggest the use of somatostatin receptor scintigraphy PET-CT for MEN-1 patients planned for duodeno-pancreatic surgery and as a useful method for surveillance if the results of the scan are going to change management (29).

**Table 5:** Current major guidelines for the use of ^68^Ga-DOTA-TATE PET/CT in the management and treatment of multiple endocrine neoplasia 1 (MEN-1) and/or neuroendocrine tumours (NET).

Society/guideline	Population to which guidelines refer to	Recommendations for use of SSTR imaging	Recommended use of SSTR imaging	Additional notes
ENETS (15, 19)	MEN-1	Yes	Should be repeated every 3 years	Recommends use of conventional imaging CT/MRI for yearly surveillance scans Role of SSTR imaging is not well defined
ESMO (32)	NETs	Yes	Whole body SSTR imaging should be part of the tumour staging, preoperative imaging and restaging	Not specific to MEN-1 Recommendation is based on high sensitivity and specificity of the modality
NANETS (USA), NCCN (USA) (17, 18)	NETs	Yes	Baseline SSTR imaging should be taken	Not specific to MEN-1 Doesn’t recommend routine use for surveillance It should only be used when there is clinical concern of disease progression which has not been demonstrated on conventional imaging modalities
International consensus statement (5)	MEN-1	Yes	For staging purposes, and to aid clinical management decisions	It can be added to surveillance of these tumours if they are noted to be growing or are >10 mm in size More prospective studies are needed to validate these recommendations

ENETS, European Neuroendocrine Tumor Society; MEN-1, multiple endocrine neoplasia-1; ESMO, European Society for Medical Oncology; NANETS, North American Neuroendocrine Tumor Society; NCCN, National Comprehensive Cancer Network; NETs, neuroendocrine tumours; SSTR, somatostatin receptor; CT, computed tomography; MRI, magnetic resonance imaging.

### Radiation risks and ^68^Ga-DOTA-TATE PET/CT

Finally, there are concerns regarding radiation doses of repeated ^68^Ga-DOTA-TATE PET/CT, especially when compared to a radiation-free modality such as MRI. Said *et al.* studied radiation risk and estimated risk of cancer death due to ^68^Ga-DOTA-TOC PET/CT and ^64^Cu-DOTATATE PET/CT in 60 patients with MEN-1. They attributed a radiation risk and estimated risk of cancer death of 0.5% during 6 years of follow-up due to SSTR-based PET-CT (30). The risk of radiation exposure in patients who are being re-staged every 2–3 years must be weighed against any possible benefits. Throughout their lifetime, patients with MEN-1, who often enter various screening regimens from the age of 10–15, might receive a cumulative radiation dose that is associated with an increased risk of iatrogenic cancer (31). Thus, the need for serial ^68^Ga-DOTA-TATE PET/CT should be carefully considered. A robust guideline or framework in patients with MEN-1 would be beneficial in highlighting which patients and at what stages the ^68^Ga-DOTA-TATE PET/CT should be done.

### Limitations

There are certain limitations to this study. First, a histopathological diagnosis was not available for all patients with an avid duodeno-pancreatic lesion on ^68^Ga-DOTA-TATE PET/CT. This meant that not all patients could be included in the calculations for sensitivity and specificity. Second, the lack of a control group meant that this study cannot draw concrete conclusions as to whether the ^68^Ga-DOTA-TATE PET/CT is beneficial compared to not having this imaging done. In addition, during the re-reading of ^68^Ga-DOTA-TATE PET/CT and CT/MRI, the re-reader was not blinded to the imaging, which could have introduced potential bias in reporting the scans as they had prior knowledge of the outcomes to a certain extent. Furthermore, there is a risk of selection bias in the study – ten patients of the 49 eligible patients were excluded because there was no record of a ^68^Ga-DOTA-TATE PET/CT on their electronic patient record. Finally, the lag time between conventional imaging and ^68^Ga-DOTA-TATE PET/CT is important; given that a few of the patients had grade 2 and 3 NETs, a potential artificial higher sensitivity of ^68^Ga-DOTA-TATE PET/CT cannot be rejected, as it could be due to natural disease progression. This study may have been more useful if ^18^F-FDG-PET/CT had also been used to see if there were any variations in the uptake of these two tracers and whether genetic factors interact with this variation or not. As ^18^F-FDG-PET/CT is not routinely performed for staging/restaging/or screening of NETs at our centre, the data regarding ^18^F-FDG-PET/CT are not available.

## Conclusions

In patients with MEN-1, ^68^Ga-DOTA-TATE PET/CT was shown to detect a greater number of duodeno-pancreatic and metastatic lesions compared to conventional cross-sectional CT or MRI imaging. Management plans were changed in most patients following their initial ^68^Ga-DOTA-TATE PET/CT. Therefore, we suggest that such somatostatin-targeted PET/CT scans should be considered in the investigation of patients with MEN-1 who are being staged for neuroendocrine tumours in order to optimise treatment outcomes.

## Supplementary materials



## Declaration of interest

The authors declare that there is no conflict of interest that could be perceived as prejudicing the impartiality of the work reported.

## Funding

This work did not receive any specific grant from any funding agency in the public, commercial, or not-for-profit sector.

## Data availability

The data that support the findings of this study are available from the corresponding author upon reasonable request.

## Ethical approval statement

As this study was a retrospective audit of practice, ethical approval was not required under the UK Policy Framework for Health and Social Care Practice. Audit registration number: CFHGCS145.
