# Supplementary material for: 68Ga-DOTA-TATE PET/CT improves accuracy and guides management in multiple endocrine neoplasia type 1 (MEN-1) patients with suspected duodeno-pancreatic neuroendocrine tumours
Source: Endocr Oncol. 2025 Sep 17;5(1):e250060. doi: 10.1530/EO-25-0060 (PMC12449681; doi:10.1530/EO-25-0060)
Supplement: Supplementary file 1 [file supplementary_materials.pdf]

Supplementary Table 1: clinical and radiological features of patients with functional Pan-NET and Gastrinoma

Age and sex	dpNET location and functionality	[68Ga] Ga-DOTA-TATE PET/CT results	Cross-sectional imaging results	Any change in the management plans following [68Ga] Ga-DOTA-TATE PET/CT
61 Male	Duodenal Gastrinoma (previously treated with surgical excision of duodenum and proximal jejunum)	No extra-pancreatic lesions seen	No extra-pancreatic lesions seen	Nil
54 Male	Pancreatic Gastrinoma	1 lesion in liver and 3 lesions in pancreas (body and tail), abnormal uptake in duodenum	1 liver lesion<br>2 cyst in pancreas<br>EUS normal, histology from EUS biopsy normal	Started on SSTAs
51 Male	Pancreatic NET, Gastrinoma	Gallium avid Aortocaval node	No extra pancreatic lesions identified	Surgical excision of gallium avid aortocaval node

47 Male	Pancreatic NET, Gastrinoma	Pituitary:1<br>Pancreas: 2<br>1 lesion adjacent to pancreas or node	1 Pituitary<br>1 Liver	SSTA started
44 Male	Pancreatic NET, Duodenal Gastrinoma	Pancreas: atleast 3<br>Duodenum: 3	None seen on CT	High doses of PPI started
52 Male	Pancreatic NET, Duodenal Gastrinoma	Pancreas: 2 areas of uptake<br>Duodenum: 1<br>Lymph nodes: 3	MRI: Pancreatic cyst, 2 nodes<br>CT: cystic pancreatic lesion	Diagnosis of MEN-1 considered based on scan results and previous history of primary hyperparathyroidism
42 Female	Pancreatic Gastrinoma	Pancreas: 2 lesions	MRI: Pancreas 2 lesions	Started on SSTAs
68 Male	Pancreatic Gastrinoma	Pancreas: 2<br>Liver: 1	MRI: liver 1 lesion	Started on SSTAs offered total pancreatectomy (not done in the end)
50 Male	Pancreatic Gastrinoma	Pancreas: 2 area of uptake, 1 cystic lesion	MRI: 1 pancreatic head cystic lesion	EUS: multiple pancreatic lesions, including head, body and tail of pancreas

71 Female	Pancreatic NET, Gastrinoma	Pancreas: 3 lesions Duodenum: 1 lesion, Left adrenal: 1	1 lesion in uncinate process, multiple lung lesions	Diagnosis of MEN-1 was considered based on Gallium DOTATATE finding and genetic test was ordered which confirmed the diagnosis
81 Female	Pancreatic NET, Insulinoma	DOTATATE avid lesion in medial half of pancreatic tail lesion	CT: soft tissue mass in tail of pancreas, cystic lesions in the head of pancreas<br>EUS couldn't be done due to hiatus hernia	Distal Pancreatectomy
55 Female	Pancreatic NET, Insulinoma	2 lesions in pancreatic body, another right paratracheal node	CT pancreas: 1 lesion	Patient referred for EUS followed by MEN-1 genetic screening, Exendin-4 scan and distal pancreatectomy
69 Female	Pancreatic NET, Insulinoma	4 pancreatic lesions, 1 duodenal lesion, 2 lymphnodes, 1 parathyroid lesion, 1 thyroid lesion	CT: 1 lesion in tail of pancreas, 1 lesion in liver, bilateral adrenal lesions	EUS and biopsy, FDG PET scan and one pancreatic lesion was non-avid on DOTATATE scan, histology confirmed NET and considered for

				surgery
58 Male	Glucagonoma	Pancreatic tail lesion	Pancreatic tail lesion	Surgery for distal pancreatic NET was offered

dpNET; duodenopancreatic neuroendocrine tumour, SSTA; somatostatin receptor analogue, NET; neuroendocrine tumour, MRI; magnetic resonance imaging, CT; computed tomography, EUS; endoscopic ultrasound, MEN-1; multiple endocrine neoplasia-1, FDG PET; fluro-deoxy-glucose positron emission tomography, PPI; proton pump inhibitors.

Supplementary Table 2: Summary of relevant findings from previous studies

Study	Comparison	Number of patients	Relevant Findings
Froeling et al. (2012)[1]	[^{68}Ga] Ga-DOTA-TOC PET/CT	• 21 patients with MEN (19 patients with MEN-1, 2 patients MEN-2)	• 10 of 21 patients (48%) had change in management plan following [^{68}Ga] Ga-DOTA-TOC PET/CT
Sadowski et al. (2015)[2]	[^{68}Ga] Ga-DOTA-TATE PET/CT vs [^{111}In] In-pentetreotide SPECT/CT vs CT	• 26 patients with MEN-1	• [^{68}Ga] Ga-DOTA-TATE PET/CT detected additional lesions in 61.5% of patients (16 out of 26 patients), not seen in other modalities<br>• [^{68}Ga] Ga-DOTA-TATE PET/CT resulted in change of management in 31% of patients (8 out of 26 patients)
Lastoria et al. (2016)[3]	[^{68}Ga]Ga-DOTA-TATE PET/CT vs CT vs EUS	• 18 patients with MEN-1	• [^{68}Ga]Ga-DOTA-TATE PET/CT had 100% sensitivity and specificity for detecting pancreatic NET in patients with MEN-1
Morgat et al. (2016)[4]	[^{68}Ga]Ga-DOTA-TOC PET/CT vs CT vs SRS with ^{111}In-pentetreotide (SRS)	• 19 patients with MEN-1	• 25 NETs seen by [^{68}Ga] Ga-DOTA-TOC PET/CT not seen by CT in blinded analysis. Un-blinded re-analysis of CE-CT images guided by [^{68}Ga] Ga-DOTA-TOC PET/CT revealed 15 of the 25 dpNETs that were not identified during blinded analysis.<br>• 13 NETS seen by CT not seen by [^{68}Ga] Ga-DOTA-TOC PET/CT<br>• 42 dpNETs seen by [^{68}Ga] Ga-DOTA-TOC PET/CT were not seen using SRS.<br>• [^{68}Ga] Ga-DOTA-TOC PET/CT detected lesions smaller

			in size than SRS
Albers et al. (2017)[5]	Screening using [^{68}Ga]Ga-DOTA-TOC PET/CT vs MRI/CT/OGD/CT	• 33 patients with MEN-1	• Investigated screening (not staging)<br>• [^{68}Ga] Ga-DOTA-TOC PET/CT detected more pancreatoduodenal lesions than MRI<br>• [^{68}Ga] Ga-DOTA-TOC PET/CT missed lesions that were detected by EUS
Kostiainen et al (2023) [6]	[^{68}Ga] Ga-DOTA-NOC PET/CT vs MRI/CT	• 58 patients with MEN-1	• Investigated screening and to characterize panNET further<br>• [^{68}Ga] Ga-DOTA-NOC PET/CT detected three times as many panNETs not visible on conventional imaging<br>• Clinical management was changed in 27 patients (47%).
Cuthbertson et al (2021)[7]	Impact of [^{68}Ga] Ga-DOTA PET/CT in familial and sporadic Pancreatic NET	• 36 patients with MEN-1 out of 42 patients with familial PanNET	• No data available specifically for patients with MEN-1.<br>• Out of 42 patients with familial PanNET, 36 patients had MEN-1<br>• Out of 42 patients with familial PanNET<br>○ 17 patients (32.7%) had identification of sites of cancer which were not previously with other imaging<br>○ In 2 patients (3.8%) ruled out metastases/disease suspected on previous imaging.<br>○ In 31 patients (59.6%) provided additional information which had impact on patient management

			◦ In 21 patients (40.4%) influenced management of patients
Mennetrey et al (2022)[8]	[68Ga]-Ga-DOTA-TOC PET/ CT	• 108 patients with MEN-1	• Comparison of [^{68}Ga] Ga-DOTA-TOC PET/CT with conventional imaging modalities in patients with MEN-1 at various stages of their disease<br>• [^{68}Ga] Ga-DOTA-TOC PET/CT was found to be superior compared to CT scan but comparable with MRI for detection of Pancreatic NET<br>• 25 patients (24%) out of 104 were offered surgical treatment following [^{68}Ga] Ga-DOTA-TOC PET/CT, whereas 3 out of 4 (75%) patients were offered PRRT following [^{68}Ga] Ga-DOTA-TOC PET/CT scans
Said et al (2023)[9]	[68Ga]-Ga-DOTA-TOC PET/CT and [64Cu]-Cu-DOTATATE PET/CT	• 60 patients with MEN-1	• Assessment of change in treatment of dpNET, bronchopulmonary NETs, thymic tumours attributed to PET/CT, to estimate radiation from imaging and risk of cancer death attributed to imaging radiation<br>• Impact on 3 out of 26 decisions to intervene in 60 patients with MEN-1<br>• Radiation risk and estimated risk of cancer death of 0.5% during 6 years follow up attributed to SRI PET/CT

References:

[1] Froeling V, Elgeti F, Maurer MH, Scheurig-Muenkler C, Beck A, Kroencke TJ, et al. Impact of Ga-68 DOTATOC PET/CT on the diagnosis and treatment of patients with multiple endocrine neoplasia. Ann Nucl Med 2012;26:738–43. https://doi.org/10.1007/s12149-012-0634-z.
[2] Sadowski SM, Millo C, Cottle-Delisle C, Merkel R, Yang LA, Herscovitch P, et al. Results of 68Gallium-DOTATATE PET/CT Scanning in Patients with Multiple Endocrine Neoplasia Type 1. J Am Coll Surg 2015;221:509–17. https://doi.org/10.1016/j.jamcollsurg.2015.04.005.
[3] Lastoria S, Marciello F, Faggiano A, Aloj L, Caracò C, Aurilio M, et al. Role of 68Ga-DOTATATE PET/CT in patients with multiple endocrine neoplasia type 1 (MEN1). Endocrine 2016;52:488–94. https://doi.org/10.1007/s12020-015-0702-y.
[4] Morgat C, Vélayoudom-Céphise F-L, Schwartz P, Guyot M, Gaye D, Vimont D, et al. Evaluation of 68Ga-DOTA-TOC PET/CT for the detection of duodenopancreatic neuroendocrine tumors in patients with MEN1. Eur J Nucl Med Mol Imaging 2016;43:1258–66. https://doi.org/10.1007/s00259-016-3319-3.
[5] Albers MB, Librizzi D, Lopez CL, Manoharan J, Apitzsch JC, Slater EP, et al. Limited Value of Ga-68-DOTATOC-PET-CT in Routine Screening of Patients with Multiple Endocrine Neoplasia Type 1. World J Surg 2017;41:1521–7. https://doi.org/10.1007/s00268-017-3907-9.
[6] Kostiainen I, Majala S, Schildt J, Parviainen H, Kauhanen S, Seppänen H, et al. Pancreatic imaging in MEN1—comparison of conventional and somatostatin receptor positron emission tomography/computed tomography imaging in real-life setting. Eur J Endocrinol 2023;188:421–9. https://doi.org/10.1093/ejendo/lvad035.
[7] Cuthbertson DJ, Barriuso J, Lamarca A, Manoharan P, Westwood T, Jaffa M, et al. The Impact of 68Gallium DOTA PET/CT in Managing Patients With Sporadic and Familial Pancreatic Neuroendocrine Tumours. Front Endocrinol (Lausanne) 2021;12. https://doi.org/10.3389/fendo.2021.654975.
[8] Mennetrey C, Le Bras M, Bando-Delaunay A, Al-Mansour L, Haissaguerre M, Batisse-Lignier M, et al. Value of Somatostatin Receptor PET/CT in Patients With MEN1 at Various Stages of Their Disease. J Clin Endocrinol Metab 2022;107:e2056–64. https://doi.org/10.1210/clinem/dgab891.
[9] Said M, Krogh J, Feldt-Rasmussen U, Rasmussen ÅK, Kristensen TS, Rossing CM, et al. Imaging surveillance in multiple endocrine neoplasia type 1: Ten years of experience with somatostatin receptor positron emission tomography. J Neuroendocrinol 2023;35. https://doi.org/10.1111/jne.13322.
